# The role of nutrition and gut microbiome in the progression of multiple myeloma and its precursor disease

**DOI:** 10.3389/fonc.2024.1461128

**Published:** 2024-10-14

**Authors:** Panagiotis T. Kanellos, Georgios K. Baxevanis, Anastasios Tentolouris, Maria Gavriatopoulou, Ioannis Ntanasis-Stathopoulos

**Affiliations:** ^1^ First Department of Propaedeutic Internal Medicine and Diabetes Center, School of Medicine, National and Kapodistrian University of Athens, Laiko General Hospital, Athens, Greece; ^2^ Department of Clinical Therapeutics, School of Medicine, National and Kapodistrian University of Athens, Alexandra General Hospital, Athens, Greece

**Keywords:** multiple myeloma, monoclonal gammopathy of undetermined significance, obesity, nutrition, gut microbiome

## Abstract

Multiple myeloma (MM) is the second most common hematological malignancy, characterized by unregulated monoclonal proliferation in the bone marrow. Monoclonal gammopathy of undetermined significance (MGUS) and smoldering multiple myeloma (SMM) are premalignant conditions that can progress to MM. Identifying etiological risk factors for MM and its precursor diseases is crucial for prevention. Obesity, diet, vitamin D levels, and gut microbiota alterations have been identified as lifestyle factors affecting MM and MGUS risk. Upon disease onset, treatment strategies aim to reduce disease burden, enhance prognosis, and optimize patients’ quality of life. Nutrition and body weight have been shown to affect disease progression and treatment outcomes. MM patients often present with vitamin D, vitamin B12, and folate deficiencies, which worsen disease prognosis. High body mass index is linked to increased death rates among MM patients and an increased risk of MGUS transformation to MM. Gut microbiota has also been associated with disease progression and response to treatment. This literature review aims to summarize the available evidence regarding the impact of nutrition and nutritional status on MM patients beyond prevention, highlighting the significance of gut microbiome and dysbiosis in MM progression.

## Introduction

Multiple Myeloma (MM) is the second most common hematological malignancy (1) which accounts for about 10% of all hematological malignancies (2, 3). The main characteristic of MM is the unregulated monoclonal proliferation in the bone marrow leading to the overproduction of nonfunctional intact immunoglobulins or immunoglobulin chains (3, 4). About 0.8% of men and women in the United States will be diagnosed with MM at some point in their life and the annual age-adjusted incidence is estimated 7.2 per 100.000 individuals between 2017 and 2021 (5).

Monoclonal gammopathy of undetermined significance (MGUS) is a premalignant asymptomatic condition where the presence of a M-protein in serum or an abnormal ratio between the free kappa and lambda light-chains (LC-MGUS) occurs, without evidence of MM or other lymphoproliferative (LP) diseases (6, 7). The prevalence of MGUS is approximately 5% in population >70 years old and increases with age (8). It is also estimated that the average annual risk of progression from MGUS to MM is about 1% (9). The prevalence of LC-MGUS, the precursor of light-chain MM, is 0.7-0.8% (10).

Identifying the etiological risk factors for MM or MGUS is of major importance for disease prevention. Several etiological risk factors related to lifestyle have been identified to alter the risk of MM or MGUS. These factors include obesity (11–17), diet (6, 18, 19), vitamin D levels (20–22) and alterations of gut microbiota (23); obesity being currently the only established modifiable risk factor (24, 25).

Upon the failure of preventive measures and the onset of disease, it becomes imperative to implement treatment strategies designed to reduce disease burden, enhance prognosis (26), and optimize patients’ quality of life (27). The introduction of new therapies has significantly enhanced the prognosis for patients with MM and have increased survival rates. However, disease progression in MM continues to result in significant morbidity rates, which becomes even more complicated by on-going treatment side-effects (28). This is closely affected by lifestyle factors such as nutrition and body weight that have been shown to have an impact on the risk of disease progression (27).

Although there are numerous studies linking nutrition and body weight with MM risk (18, 29–32), the available data on the effect of these aspects on disease progression is limited (27). Over the last decades a part of research has been focusing on how nutritional aspects and body weight modulate the active MM disease course as well as their effect on treatment (27). Studies have shown that MM patients present vitamin D, vitamin B12 (33) and folate (33, 34) deficiencies, worsening disease prognosis. Additionally, high body mass index (BMI) is linked with higher death rates among MM patients (35) as well as with a higher risk of progression of MGUS to MM (36). Gut microbiota has also been the subject of research with studies showing an association between microbiota and disease progression-response to treatment (37–39).

The purpose of this literature review is to summarize and elucidate the available evidence about the impact of nutrition and nutritional status on MM patients, beyond the context of prevention, in an effort to highlight its significance on disease prognosis and emphasize on the role of gut microbiome and dysbiosis in MM progression.

## Epidemiology

Multiple Myeloma represents about 1% of all cancers and roughly 10% of all hematologic malignancies (40). Over the last decades, an increased number of global deaths has been reported due to MM, with limited information regarding epidemiology and disease burden especially in developing countries (41). It has been estimated that each year about 32.000 individuals are diagnosed with the disease and about 13.000 lose their life (42). Between 2017 and 2021, MM presented an annual age-adjusted incidence of 7.2 per 100.000 individuals (5). According to a study conducted by Cowan et al. (2018), MM incident cases from 1990 to 2016 increased by 126%, and deaths increased by 94%. Of the 126% increase in global incident cases, 40.4% was due to population growth, 52.9% was attributed to an aging population, and 32.6% resulted from higher age-specific incidence rates (41). MM appears to have different incident rates between sexes with men having slightly higher incident rates than women. Studies have also shown that there are racial disparities, with Afro-descendants presenting two times higher risk compared to their White counterparts (40) and Asians presenting lower incidence compared to Whites. MM incidence depends on age and reaches its maximum during the seventh decade of life (43). The median age of patients at the time the diagnosis is 65 years (44), with very few cases occurring under the age of 40 (43). Additionally, randomized controlled trials using modern therapy have found a median survival of MM patients of approximately 6 years (45).

Regarding the premalignant conditions, MGUS presents an overall prevalence of 2.4% (46) and 70 years mean age at the time of diagnosis, being predominantly a disease of the elderly (46, 47). Similarly to MM, MGUS is also associated with race and ethnicity (48). The highest age-adjusted prevalence of MGUS is reported in the black population (0.99%), followed by Mexican Americans (0.55%), while the lowest prevalence is observed in white populations (0.21%) (49). Concerning smoldering multiple myeloma (SMM), the prevalence of this premalignant condition is 0.5% in individuals over 40 years of age and increases with age while it is higher in men than in women (0.7% vs. 0.4%) (50). Similar to MGUS, SMM also presents race disparities with high prevalence in black individuals than other racial groups (51–53). However, it should be noted that because of the asymptomatic nature of SMM its diagnosis is rare, and it is estimated that only 3-6% of patients with MM are diagnosed at this precursor state (54, 55).

## The impact of body composition on multiple myeloma progression

Obesity is a well-established and potentially modifiable risk factor linked to a higher incidence of MM (56, 57). However, the association of obesity with clinical outcomes after MM diagnosis is less clear.

Data from two pooled analyses of large prospective cohorts have indicated a relation between high BMI and mortality. However, it remains vague whether increased cancer incidence, decreased survival after diagnosis, or both may explain the increased mortality among patients with higher BMI. A pooled analysis of MM mortality involving 1.5 million participants (including 1,388 MM deaths) from 20 prospective cohorts within the National Cancer Institute Cohort Consortium observed an increased MM mortality associated with higher BMI in early adulthood, as well as higher BMI and waist circumference at cohort entry (16). Moreover, data from seven prospective cohorts including 239.597 African Americans adults indicated a steady increase in mortality with rising BMI. The hazard ratios reached 1.43 (95% CI: 1.03 to 1.97) for BMIs of 35 kg/m² or higher compared to those with normal BMIs (58).

Another study involving 2,968 MM patients in the Veterans Health Administration System found that underweight patients had increased mortality, while overweight and obese patients had lower mortality compared to those with normal weight. Additionally, patients with weight loss of 10% or more from baseline in the year prior to diagnosis indicated increased mortality (59).

In a subgroup of 108 patients who underwent a whole-body low-dose computed tomography before induction therapy, a significant inverse correlation was observed between adverse cytogenetics and both visceral adipose tissue (VAT) of the abdomen and pelvis. No correlation was found between visceral or subcutaneous adipose tissue and adverse events. However, a significant inverse correlation was observed between abdominal (p=0.03) and pelvic (p=0.035) VAT and treatment response. Abdominal VAT remained significant (p=0.034) independently of revised ISS stage and treatment. BMI did not show a significant correlation with treatment response or the investigated cytogenetics (60).

A very recent study examined the impact of BMI on progression-free survival (PFS) and overall survival (OS) in 1,142 newly diagnosed patients from the Multiple Myeloma Research Foundation CoMMpass registry. The results showed that underweight and severely obese patients had lower median PFS and OS compared to those who were normal weight, overweight or moderately obese. Multivariable models linking PFS and OS with BMI indicated that underweight patients had a significantly higher risk of death (HR: 2.32; 95% CI: 1.09, 4.97) (61).

In addition, apart from body weight per se, measures of muscle and adipose tissue mass have been associated with poor outcomes in various malignancies (62). A study examined the association between muscle and fat areas and radiodensity, and OS in people with newly diagnosed MM (62). Overall, 341 patients diagnosed with MM from 2010-2019 who had an 18F-fluorodeoxyglucose positron emission tomography/computed tomography within 3 months prior to diagnosis, or after diagnosis but within <1 month of starting treatment were included in the study. Median follow up was 5.7 years. Sarcopenia was defined as skeletal muscle index below the sex-specific median in the population. The prevalence of sarcopenia ranged from 46% to 56% depending on the cutoff used. Median PFS was 33.4 (95% CI 27.2-49.8) months in patients with sarcopenia compared to 45.0 (95% CI: 31.9-71.0) months in patients without sarcopenia (p=0.25). Median OS was 7.6 (95% CI: 5.8-not reached) years and 9.3 years (95%CI: 6.1-not reached) in patients with and without sarcopenia, respectively (p=0.77). Nevertheless, low muscle radiodensity was associated with higher disease stage, anemia, and renal failure. OS was 5.6 vs. 9.0 years in patients with muscle radiodensity in the lower vs. middle/upper tertiles, respectively (p=0.02). Authors conclude that body composition evaluating using routine low-dose CT images taken at diagnosis can offer valuable prognostic insights for patients with MM. Assessments of muscle and fat quality might be more effective in predicting disease outcomes than simply measuring muscle and fat quantity (62).

Another study also examined whether the presence of sarcopenia had prognostic value in patients with newly diagnosed MM (63). Sarcopenia was determined by utilizing a deep learning-based convolutional neural network algorithm on CT images of the abdomen. Subjects with newly diagnosed MM from January 2005 to July 2019 who had a standard dose CT scan that included the L3 vertebral level performed anytime within 6 months of diagnosis were enrolled in the study. A total of 322 participants were included in the analysis. The median age was 66 (range 37-95) and 20% were older than 75 years of age. The median BMI was 26.7 (range: 16.2-59.7) and 30% had a BMI greater than 30. Of the 200 patients with FISH cytogenetics information available, 49 (25%) were categorized as having high-risk cytogenetics. The median follow-up for this cohort was 72 months (95% CI: 63-83). Overall, 171 (53%) patients in this study cohort were categorized as sarcopenic. The study showed that the median OS for sarcopenic patients was 45 months compared to 90 months for those not sarcopenic (p=0.0005). Moreover, the 2-year mortality rate for sarcopenic patients was 40% compared to 18% for the non-sarcopenic patients (p<0.0001). In the multivariable model, the adverse prognostic impact of sarcopenia was independent of International Staging System (ISS) stage, age, and high-risk FISH cytogenetics (63).Therefore, body composition may be associated with poor outcomes in people with MM. However, further prospective studies are needed to examine whether sarcopenia is independently associated with MM outcomes.

## Nutrition as a risk factor for multiple myeloma

The etiology of MGUS remains largely unknown and there have been no studies examining the impact of diet on MGUS or its progression to MM. Data from the population-based AGES Study (N=5,764) indicated that fruit consumption at least three times per week during adolescence led to lower risk of MGUS (OR=0.62, 95%CI 0.41-0.95) and fruit consumption at least three times per week during the late life led to decreased risk of progressing from MGUS to MM (HR=0.34, 95%CI 0.13-0.89) when compared to lower fruit consumption.

Additionally, limited studies have evaluated the relation between diet and MM, with the results being inconclusive. Regarding meat consumption, one older study found a significant increase in risk per portion of red meat daily (64), while another one found that meat consumption increased non-significantly the risk among Blacks and decreased non-significantly the risk among Whites (18). Three studies have found an inverse association of fish intake and risk of MM (18, 19, 65). Moreover, two studies have examined dairy product consumption: one reported increased risk for total dairy intake and eggs (18) while the other identified a significant increased risk linked to yogurt consumption (66). In another study, vegetables consumption was found to have an inverse association with MM and a non-significant increase in risk for fruit consumption (18). In a more recent population-based case-control study, inverse associations for cooked tomatoes, cruciferous vegetables, fresh fish, alcohol and vitamin A and positive associations for cream soups, jello, ice cream and pudding with MM have been observed (30).

In a very recent study, after analyzing data from prospective cohorts of 69751 women (Nurses’ Health Study, 1984-2014) and 47232 men (Health Professionals Follow-up Study, 1986–2014), the association between dietary pattern and risk of MM was examined. In men, empirical dietary inflammatory pattern, empirical dietary indices for insulin resistance and empirical dietary indices for hyperinsulinemia were statistically significantly associated with increase in MM risk (16%, 9% and 11%, respectively) (33).

## The impact of nutrition on multiple myeloma and the nutritional status of patients

Nutrition is considered as a major modifiable risk factor for cancer and MM development with many available studies assessing the relation between nutrition and MM risk (18, 30, 61, 67). However, studies about the role of nutrition and nutritional status on MM patients and disease progression are limited (27) (**Table 1**).

**Table 1:** Characteristics of studies included and their pertinence to nutrition.

Study	Study design	Characteristic examined	
Lee et al., 2020 (68)	Prospective analysis	Dietary patterns	AHEI-2010, aMED, DASH, Prudent, Western, EDIP, EDIR, EDIH
Sergentanis et al., 2018 (69)	Meta-analysis	Food groups	Fruits, vegetables
Borsi et al., 2021 (70)	Cross-sectional	Macronutrients	Carbohydrate
Hoogstraten et al., 1965 (71)	Cross-sectional	Micronutrients	Folate
Hoffbrand et al., 1967 (72)	Cross-sectional	Micronutrients	Folate, Vitamin B12
Greenfield et al., 2014 (33)	Cross-sectional	Micronutrients	Vitamin D, Folate, Vitamin B12
Ng et al., 2009 (76)	Cohort	Micronutrients	Vitamin D
Badros et al., 2008 (77)	Cohort	Micronutrients	Vitamin D
Wang et., 2016 (78)	Non-randomized clinical trial	Micronutrients	Vitamin D
Lauter et al., 2015 (79)	Retrospective cohort	Micronutrients	Vitamin D
Oortgiesen et al., 2023 (80)	Prospective, single-arm	Micronutrients	Vitamin D
Ismail et al., 2023 (81)	Meta-analysis	Micronutrients	Vitamin D

AHEI, alternate healthy eating index-2010; aMED, alternate Mediterranean diet; DASH, dietary approaches to stop hypertension; EDIH, empirical dietary index for hyperinsulinemia; EDIP, empirical dietary inflammatory pattern; EDIR, empirical dietary index for insulin resistance.

### Dietary patterns, food groups and macronutrient intake

A recent pooled prospective survival analyses of 423 MM patients derived from the Nurses’ Health Study (1986-2016) and the Health Professionals Follow-up Study (1988-2016) examined the association of dietary patterns (Alternate Healthy Eating Index (AHEI)-2010, Alternate Mediterranean Diet, Dietary Approaches to Stop Hypertension, Prudent, Western and empirical dietary inflammatory patterns and empirical dietary indices for insulin resistance and hyperinsulinemia) followed before diagnosis with MM survival. Data showed 295 deaths related to MM among 345 total deaths. Mortality was 15-24% lower for each one standard deviation (SD) increase in favorable dietary pattern scores and 16-24% higher per 1-SD increase in “unhealthy” diet scores. Researchers concluded that MM patients with healthier dietary habits before diagnosis may exhibit better survival than those who followed less healthy dietary patterns (68).

A recent systematic review examined the association between fruit and vegetable consumption with the risk of hematological malignancies. Neither fruit nor vegetable consumption was related to MM risk (69).

A very recent study assessed the knowledge about nutrition and the quality of diet in 61 patients with MM. Significant changes of clinical parameters (hemoglobin, uric acid, albumin, total proteins, beta-2 microglobulin, percentage of plasmacytes in the bone marrow and D-dimers) were observed in patients with high or low carbohydrate intake compared to patients with medium carbohydrate consumption. Moreover, their knowledge about nutrition was not associated either with clinical indicators of disease status nor with their nutrient intake (70).

### Micronutrients

It has long been known that MM patients may present nutritional deficiencies especially on folate levels. Early enough, in 1965, a study of Hoogstraten et al. showed that patients with active MM presented lower serum folate levels (71). Another study of Hoffbrand et al. (1967) aiming to determine the incidence, severity, and cause of B12 deficiency in patients with MM showed that among 32 patients participating in the study, 26 patients had low serum folate levels and 5 had vitamin B12 deficiency (72). In a more recent study aiming to evaluate the body composition and the nutritional status, among other parameters, of 32 intensively treated patients, with advanced but stable MM, 25% of the patients had reduced serum folate, 6% had reduced vitamin B12 and 59% of patients were vitamin D insufficient/deficient (33). Vitamin D levels have been of concern to the scientific community for its involvement in various diseases (73). MM is the most common malignancy affecting bone and up to 90% of patients develop bone lesions (74). On that account, studies have examined the prevalence of vitamin D deficiency in MM individuals and its role in disease progression (33, 75, 76). In a study conducted in 148 newly diagnosed MM patients, a ‘‘step-wise’’ association between International Staging System (ISS) staging and vitamin D deficiency was found. Additionally, the prevalence of vitamin D deficiency increased in parallel with ISS and reached its peak in stage 3 with 37% of patients presenting deficiency (76). It is also of major importance that subjects presenting vitamin D deficiency also present higher serum CRP and creatinine levels, markers that have been shown to predict MM prognosis, with the authors suggesting that vitamin D deficiency may lead to lower MM prognosis due to its activity in both skeletal and non-skeletal level (76). Greenfield et al. confirmed these results showing that 59% of the patients participating in the study were vitamin D insufficient/deficient (33). The high incidence of low vitamin D levels in MM patients compared to the general population, is predominantly caused by lower levels of activity in MM patients resulting in low sunlight exposure and it is independent of age, sex and disease activity (77). Recently, studies have highlighted that treatments such as thalidomide or bortezomib may be additional factors contributing to vitamin D deficiency and this is also associated with peripheral neuropathy. Moreover, vitamin D supplementation may prevent the development of severe neuropathy or reduce its severity among patients with MM (78). A study aiming to evaluate serum 5-hydroxyvitamin D (25(OH)D) status and supplementation with vitamin D on MM patients found that supplementation led to significantly higher levels of (25(OH)D) (79). Also, hemoglobin, leukocyte, erythrocyte levels were higher after supplementation, while lower thrombocyte levels were found. However, the authors concluded that although significant serum (25(OH)D) increases were observed, sufficient levels were not achieved even with doses exceeding the 1000 IUs per day (79). Contrary to these results, in a more recent study vitamin D supplementation of MM patients using substantially higher doses than recommended in guidelines, and more specifically a 3-level dose escalation regimen with a loading dose of 200,000 IU given at the beginning of the study and a maintenance dose of 800 IU, has been proved to be an effective approach to achieving adequate vitamin D levels after 6 months of supplementation. It has to be noted that the low toxicity risk allowed the use of high doses of the vitamin D supplementation (80). However, it is evident that more studies are needed to assess the efficacy of vitamin D supplementation on serum 25(OH)D levels and clarify the efficacy of such approaches and establishing specific guidelines for the repletion of not only 25(OH)D levels but also other vitamins through supplementation in patients with MM appears to be a distant goal.

A very recent meta-analysis which included 18 studies found that the prevalence of serum vitamin D deficiency and insufficiency in patients with MM was 39.4% and 34.1%, respectively. It was also observed a greater proportion of newly diagnosed patients who exhibited vitamin D deficiency and insufficiency (43.0% and 41.6%, respectively), compared to patients who were under treatment (41.6% and 32.3%, respectively) (81).

### The role of gut microbiota in the progression of multiple myeloma

The human microbiota is a distinct collection of microorganisms that inhabit and evolve within the human body from the onset of life. These microorganisms interact with the host’s immune system, aiding in the formation of defenses against pathogens. Gut microbiome alterations, namely intestinal dysbiosis, are closely related to a variety of diseases. However, the mechanisms through which commensal bacteria influence a broad range of mucosal and extramucosal human disorders have only been partially understood. So far, little is known about the role of the gut microbiome and alterations of its metabolic functions in the development of MM (82).

In a cohort aiming to investigate the potential relation between the gut microbiome and MM, researchers found that higher bacterial diversity was associated to significant differences in metagenomic composition in 19 patients with newly diagnosed MM. Eleven opportunistic pathogens (*Raoultella ornithinolytica, Citrobacter freundii, Enterobacter cloacae, Klebsiella aerogenes, Klebsiella variicola, Klebsiella pneumoniae, Streptococcus salivarius, Streptococcus oralis, Streptococcus gordonii, Streptococcus mitis, and Streptococcus pneumoniae*) were found to have higher relative abundance in MM patients; these bacteria are nitrogen-recycling and may result from the accumulated urea nitrogen in MM. Moreover, fecal microbiota transplantation into 5TGM1 mice propose that a possible mechanism for the interaction between MM-enriched bacteria and MM progression is recycling urea nitrogen. Furthermore, MM progression seems to be promoted by *Klebsiella pneumoniae* via *de novo* synthesis of glutamine in mice while progression of MM was found milder in the mice fed with glutamine-deficient diet (83).

In a very recent study, the association of sustained minimal residual disease (MRD) negativity with dietary factors, stool metabolites and the stool microbiome in MM patients on lenalidomide maintenance was examined. Patients with sustained MRD negativity had increased stool butyrate amount, higher relative abundance of predicted butyrate producers and higher alpha diversity of the fecal microbiome at 3 months. Additionally, dietary proteins from seafood and plants were associated with butyrate at 3 months and sustained MRD negativity. Consumption of anthocyanidins, flavones, and flavanols was also associated with stool butyrate concentration (84).

In a very recent study, the gut microbiome and dietary intake in 30 patients with MM undergoing autologous stem cell transplantation were evaluated. A loss of alpha-diversity at the timepoint of engraftment due to a decrease in *Blautia*, *Ruminococcus*, and *Faecalibacterium* genera as a consequence to intravenous antibiotic exposure was observed. Alpha-diversity is a numerical measure used in microbiome studies to summarize species abundance within a sample, taking into account both species richness (number) and evenness (distribution). Higher alpha-diversity often indicates a healthier microbiota. Moreover, patients with higher fiber intake indicated higher relative abundance of *Blautia* at the pre-transplant timepoint. Partial response to therapy compared with complete response or very good partial response was observed to patients with lower alpha-diversity at engraftment timepoint (85).

Overall, the results of the aforementioned studies reveal a new role of the altered gut microbiome in the progression of MM suggesting new treatments by influencing the gut microbiota of patients with MM.

## Conclusion

While there is rising interest in the role of diet and the gut microbiota in the development of MGUS and SMM to symptomatic myeloma, it is critical to show caution when drawing causal inferences. Diet, microbiota and cancer progression exhibit complicated interactions that are regulated by a variety of factors, including genetics, lifestyle and environmental exposure. Most existing studies are observational, making it difficult to establish direct causation. Therefore, while dietary interventions may show promise, they should be considered as part of a broader, multidimensional approach to disease management. Further prospective research examining longitudinal outcomes is necessary to determine the precise impact of dietary patterns, the interplay with microbiota and the role of dietary interventions on myeloma progression.
